# Associations of Functional Dyspepsia with Eating Behaviors and Stress-Coping Styles Among Japanese University Students

**DOI:** 10.3390/nu18091316

**Published:** 2026-04-22

**Authors:** Yoshie Miyake, Koki Takagaki, Atsuo Yoshino, Toru Hiyama, Yuri Okamoto

**Affiliations:** Health Service Center, Hiroshima University, 1-7-1, Kagamiyama, Higashi-Hiroshima, Hiroshima 739-8514, Japan

**Keywords:** functional dyspepsia, eating behaviors, depressive symptoms, stress-coping styles, university students

## Abstract

**Background/Objectives:** Functional dyspepsia (FD) is relatively common among young adults and is increasingly understood within the framework of brain–gut interactions. Eating behaviors and psychological distress may be related to FD, but evidence in young adults remains limited. This study examined the associations between FD and eating behaviors and depressive symptoms among university students. **Methods:** A cross-sectional study was conducted during health checkups. A total of 4328 students (2232 males and 2096 females) completed questionnaires assessing FD symptoms based on Rome IV, eating behaviors (EAT-26 and BITE), depressive symptoms (BDI-II), and coping styles (CISS). We compared scores between students with and without FD and performed multivariable logistic regression including gender, BMI, sleep, eating behaviors, and depressive symptoms. **Results:** The prevalence of questionnaire-based FD was 6.1% in males and 7.2% in females. Students with FD had higher EAT-26, BITE, BDI-II, and emotion-oriented coping scores. In multivariable logistic regression, EAT-26 ≥ 10 (OR: 1.92, 95% CI: 1.26–2.91, *p* = 0.002), BITE ≥ 10 (OR: 1.45, 95% CI: 1.01–2.08, *p* = 0.04), BDI-II ≥ 10 (OR: 3.83, 95% CI: 2.97–4.95, *p* < 0.001), and BMI < 18.5 kg/m^2^ (OR: 1.74, 95% CI: 1.31–2.31, *p* < 0.001) were significantly associated with FD; gender and sleep were not. **Conclusions:** FD was associated with disordered eating behaviors, depressive symptoms, and low BMI. Differences in emotion-oriented coping were observed between groups. These findings suggest that integrating assessments of gastrointestinal symptoms, eating behaviors, and psychological factors may inform early detection and support at university.

## 1. Introduction

Functional dyspepsia (FD) is a functional gastrointestinal disorder (FGID) with a high global prevalence, and it impairs patients’ quality of life [1]. FD is characterized by persistent or recurrent upper abdominal pain or discomfort in the absence of a peptic ulcer or organic disease [2,3], and it is increasingly understood within the framework of brain–gut interactions. Diagnosis is based on the Rome IV criteria [4]. A recent global epidemiological study reported the Rome IV-based FD prevalence of 7.2% with considerable regional variation ranging from 2.44% in Japan to 12.28% in Egypt; prevalence also decreases with age [5]. Although previous studies have reported that FD is relatively common among young adults [1,6], most epidemiological evidence has been derived from studies of the general adult population, and there are relatively few studies focused specifically on young people. Evidence regarding FD among university students is still limited.

FD is closely linked to psychological and behavioral factors. Patients with FD often report poorer health status, mental health, and social functioning than do those with structural gastrointestinal pathology [7,8]. Many patients recognize meals as the main triggering factor, but quality evidence concerning the relationship between specific foods and the onset of FD symptoms is scarce [9]. A previous study revealed a positive association between FD and irregular eating patterns [10]. Dietary restrictions, irregular meal timing, or altered eating patterns may be caused by persistent FD symptoms. Recently, there has been increasing awareness of the significance of symptoms related to gut–brain interaction disorders (DGBIs) in patients with eating disorders (EDs) [11,12]. Previous studies have reported a high incidence of FD in patients with EDs and stress-associated diseases [13,14,15,16,17]. EDs are characterized by aberrant eating patterns and impairments in emotional processing [18]. Altered eating behavior is strongly associated with disturbed gastrointestinal sensitivity and motor physiology [13,17]. Patients with EDs present with various gastrointestinal disturbances, such as postprandial fullness, abdominal distention, abdominal pain, gastric distension, and early satiety [19]. Disordered eating behaviors, both clinical and subthreshold, have been significantly associated with poor psychological and physical outcomes [20,21], and the prevalence of EDs has increased among university students. Once established, psychological and physiological disturbances can reinforce each other, resulting in a FGID that can persist independently of the ED that originally caused the motor and sensory abnormalities [13]. However, few studies have examined the association between FD and disordered eating behaviors among university students.

The pathophysiology of FD is multifactorial [22], and psychosocial factors such as depressive symptoms, anxiety and impaired stress coping are considered to play important roles in the onset and maintenance of symptoms [23,24,25,26,27]. In particular, stress has been identified as a highly sensitive and specific predictor and is an important factor in the pathogenesis of FD, increasing duodenal permeability by activating mast cells through corticotropin-releasing hormone [27,28,29,30]. Several studies have suggested that psychological stress can modulate gut function and lead to gastrointestinal disorders [28,29,30]. Stress-coping behaviors have been linked to the development of stress-related diseases [31], and coping strategies used to manage stress vary from individual to individual [32,33]. If coping strategies are inappropriate or insufficient to minimize the threat, it may lead to psychosomatic responses [34]. Psychological interventions could be beneficial for patients with FD [8,35,36,37,38]. Nevertheless, the stress-coping styles among university students with FD have not been well characterized.

The purpose of this study was to investigate the associations between FD and eating behaviors and depressive symptoms among Japanese university students. Understanding the psychological and behavioral correlates of FD in university students may contribute to improving screening and support approaches in university health services. We hypothesized that students with FD would have higher rates of disordered eating behaviors, depressive symptoms, and maladaptive coping styles compared with those without FD.

## 2. Materials and Methods

### 2.1. Participants

Participants were university students at Hiroshima University. In the 2023 academic year, the total number of students enrolled at the university was 10,558, of whom 6811 attended the annual health checkup. Health checkups are not compulsory at our university. The questionnaire survey was conducted among students attending the health checkup, and participation in the questionnaire was also voluntary. As part of the health checkup, students were asked to complete self-report questionnaires. Students who did not complete the questionnaires or had missing data in key variables were excluded from the study.

### 2.2. Procedure

Participants were classified as having FD or non-FD according to the Rome IV criteria based on self-reported gastrointestinal symptoms. Differences in eating behaviors, depressive symptoms, and coping styles were examined between students with and without FD. Eating attitudes were assessed using the Eating Attitudes Test-26 (EAT-26) [39]. Participants were classified as having disordered eating attitudes (clinical or subthreshold; score ≥ 10) or non-disordered eating attitudes (score ≤ 9). In addition to the conventional cutoff score of ≥20 indicating clinically significant eating disorders, a cutoff score of ≥10 was used to identify moderate or subthreshold disordered eating attitudes in this student population, based on previous studies [20,40,41]. Bulimic symptoms were assessed using the Bulimic Inventory Test, Edinburgh (BITE) [42]. Participants were classified as having bulimic symptoms (clinical or subthreshold; score ≥ 10) or non-bulimic symptoms (score ≤ 9) on the basis of previous studies [42]. Depressive symptoms were assessed using the Beck Depression Inventory-II (BDI-II) [43]. Participants were classified as having depressive symptoms (clinical or subthreshold; score ≥ 10) or non-depressed (score ≤ 9) on the basis of previous studies [44,45]. We compared the frequencies of the two categories of disordered eating attitudes, bulimic symptoms and depressive symptoms, between students with and without FD. The questionnaires were administered as part of an annual checkup. Informed consent was obtained in opt-out form. An opt-out informed consent protocol was used for the use of participant data for research purposes. The study protocol was reviewed and approved by the Ethics Committee of the Hiroshima University School of Medicine, Japan (approval number E-2019-1767) and was conducted ethically in accordance with the Declaration of Helsinki.

### 2.3. Questionnaires

The questionnaire consisted of three sections. The first section collected the characteristics of the participants, including age, sex, body mass index (BMI), and average sleep duration. The second section assessed gastrointestinal symptoms using a questionnaire based on the Rome IV criteria for FD. The third section evaluated eating behaviors and psychological factors, including EAT-26, BITE, BDI-II, and the Coping Inventory for Stressful Situations (CISS) [46].

#### 2.3.1. Assessment of FD

Participants were classified as having questionnaire-based FD based on self-reported gastrointestinal symptoms using the Rome IV criteria [4,47]. Clinical assessment or endoscopic evaluation was not performed. According to recent Japanese guidelines on FD, self-report questionnaires are useful for identifying FD [47]. FD was defined as any combination of the following four symptoms persisting for the last three months with symptom onset at least six months prior to diagnosis: (1) bothersome postprandial fullness, (2) bothersome early satiation, (3) bothersome epigastric pain, or (4) bothersome epigastric burning.

#### 2.3.2. Eating Attitudes Test-26

The EAT-26 is a 26-item self-report questionnaire that is used to assess eating attitudes on a 6-point scale ranging from “not at all” to “extremely” [39]. Cronbach’s alpha coefficients ranged from 0.85–0.94 [48]. The Japanese version has demonstrated high reliability and validity [49,50]. Buddeberg-Fischer et al. [40] have suggested that EAT-26 scores between 10 and 19 may indicate moderate levels of disordered eating behaviors. In accordance with previous studies [40], participants were categorized into severely disordered (≥20), moderately disordered (10–19), and non-disordered (0–9) groups. A score greater than 9 indicates the presence of severe or moderate disordered eating attitudes.

#### 2.3.3. Bulimic Inventory Test, Edinburgh

The BITE is a self-report measure of bulimic symptoms and consists of a 30-item symptom scale and a 6-item severity scale [42]. The symptom evaluation scale is scored as yes or no, and the scores range from 0 to 30. The cutoff point suggested for the symptom subscale was 20 points [42]. Cronbach’s alpha coefficient was 0.96. The Japanese version has shown high reliability and validity [51]. Clinical bulimic pathology is identified by a cutoff point of 20. A score of 10 or above reflects a subclinical group of subjects. In accordance with previous studies [42], participants were categorized into clinical (≥20), subthreshold (10–19), and non-bulimic (≤9) groups. A score greater than 9 indicates the presence of clinical or subthreshold bulimic symptoms.

#### 2.3.4. Beck Depression Inventory II

The BDI-II is used to measure depressive symptoms, and it consists of 21 self-reported items rated on a 4-point scale [43]. The cutoff point for clinical depression is a score of 18 on the BDI-II. A cutoff score of 18 yielded a sensitivity of 94% and a specificity of 92%, and a cutoff score of 10 yielded a sensitivity of 100% and a specificity of 70% [44]. Cronbach’s alpha coefficient was 0.87 [52]. The Japanese version has demonstrated high reliability and validity [53]. In accordance with previous studies [44,45], participants were categorized into clinical (≥18), subthreshold (10–17), and non-depressed (≤9) groups. Subthreshold depression is defined as clinically significant depressive symptoms [44,45]. A score greater than 9 indicates the presence of clinical or subthreshold depressive symptoms.

#### 2.3.5. Coping Inventory for Stressful Situations

The CISS is a 48-item self-report measure scored on a 5-point scale that consists of three subscales that are used to evaluate coping behaviors: task-oriented (CISS-T: solving a problem, cognitive restructuring of a problem, or attempts to alter a situation), emotion-oriented (CISS-E: emotional responses to a problem), and avoidance-oriented (CISS-A: seeking distractions) coping [46]. Cronbach’s alpha coefficients range from 0.75–0.89, and the Japanese version has demonstrated high reliability and validity [54,55].

### 2.4. Data Analysis

Statistical analyses were performed via SPSS version 28 (IBM Corp., Armonk, NY, USA). Participant characteristics were averaged. We used independent-samples t-tests to compare the EAT-26, BITE, BDI-II and CISS scores between students with and without FD. Next, chi-square and residual analyses were used to compare the frequencies of disordered eating attitudes, bulimic symptoms, depressive symptoms and sleep duration between students with and without FD. For logistic regression analysis, EAT-26, BITE, and BDI-II scores were dichotomized using cutoff values reported in previous studies to identify students with clinically relevant or subthreshold levels of disordered eating behaviors and depressive symptoms. A multivariable logistic regression analysis was conducted to examine factors associated with FD among participants (gender: female; low BMI: BMI < 18.5 kg/m^2^; sleep duration ≤ 6 h/day; disordered eating attitudes: EAT-26 score ≥ 10; bulimic symptoms: BITE score ≥ 10; depressive symptoms: BDI-II score ≥ 10). To examine potential gender differences, we additionally tested interaction terms between gender and each predictor variable in the logistic regression model. Statistical significance was set at *p* < 0.05.

## 3. Results

### 3.1. Participant Characteristics

**Figure 1:**
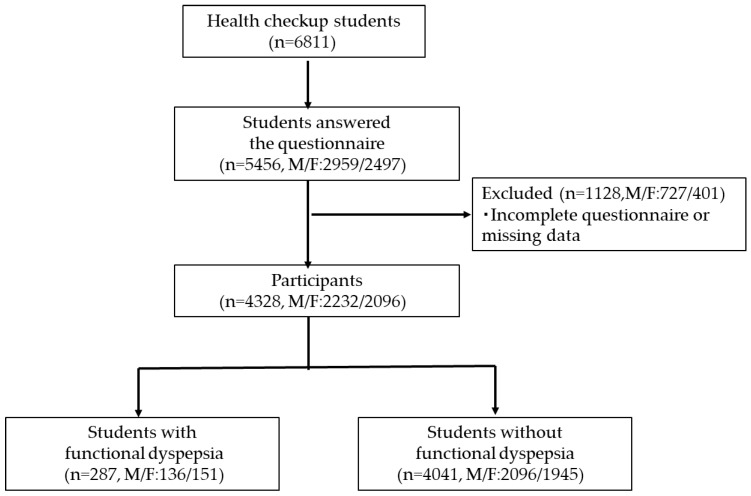
Participant flow chart.

Of the 6811 students who attended the health checkup, 5456 students (2959 males; mean age: 20.0 ± 2.7 years, and 2497 females; mean age: 19.8 ± 1.8 years) responded to the questionnaire. After excluding students with incomplete questionnaires or missing data in key variables, 4328 students (2232 males; mean age: 20.1 ± 2.7 years, and 2096 females; mean age: 19.8 ± 1.6 years) were included in the final analysis (Figure 1). The prevalence of questionnaire-based FD was 6.1% (*n* = 136) in males and 7.2% (*n* = 151) in females. There was no significant difference in FD prevalence between males and females (χ^2^(1) = 2.15, *p* = 0.14). Among females, students with FD scored a significantly lower BMI than those without FD (*p* < 0.05). Most students reported sleeping 6–7 h per night. No significant differences were observed in sleep duration between students with and without FD. The results of the annual checkup are shown in Table 1.

### 3.2. Questionnaire Results

The results of the questionnaires are shown in Table 2. In males, students with FD scored significantly higher on the EAT-26 (*p* < 0.001), BITE (*p* < 0.001), BDI-II (*p* < 0.001) and CISS-E (*p* < 0.01) than did those without FD. In females, students with FD scored significantly higher on the EAT-26 (*p* < 0.001), BITE (*p* < 0.001), BDI-II (*p* < 0.001), and CISS-E (*p* < 0.001), and significantly lower on the CISS-T (*p* < 0.05) than did those without FD.

### 3.3. Frequencies of Disordered Eating Behaviors

With respect to eating attitudes, among male students with FD, 10.3% were classified as having disordered eating attitudes, compared with 3.1% of those without FD. Among female students with FD, 16.6% were classified as having disordered eating attitudes, compared with 5.9% of those without FD. The chi-square test revealed significant differences between students with and without FD (χ^2^(1) = 18.86, *p* < 0.001 for males and χ^2^(1) = 25.88, *p* < 0.001 for females). The residual analysis revealed that the number of participants in the category of disordered eating attitudes was significantly greater among students with FD (males: adjusted standardized residual, asr = 4.3; females: asr = 5.1). The results are shown in Table 3.

With respect to bulimic symptoms, among male students with FD, 11.8% were classified as having bulimic symptoms, compared with 5.6% of those without FD. Among female students with FD, 28.5% were classified as having bulimic symptoms, compared with 13.2% of those without FD. The chi-square test revealed significant differences between students with and without FD (χ^2^(1) = 8.72, *p* < 0.01 for males and χ^2^(1) = 26.87, *p* < 0.001 for females). The residual analysis revealed that bulimic symptoms were significantly more frequent among students with FD (males: asr = 3.0; females: asr = 5.2). The results are shown in Table 3.

### 3.4. Frequencies of Depressive Symptoms

Among male students with FD, 47.1% were classified as having depressive symptoms, compared with 16.4% of those without FD. Among female students with FD, 45.7% were classified as having depressive symptoms, compared with 17.3% of those without FD. The chi-square test revealed significant differences between students with and without FD (χ^2^(1) = 80.29, *p* < 0.001 for males and χ^2^(1) = 72.20, *p* < 0.001 for females). The residual analysis revealed that the number of depressed participants was significantly greater among students with FD (males: asr = 9.0; females: asr = 8.5). The results are shown in Table 4.

### 3.5. Factors Associated with FD

In multivariable logistic regression analysis, the factors significantly associated with FD were BMI < 18.5 kg/m^2^ (odds ratio (OR): 1.74, 95% CI: 1.31–2.31, *p* < 0.001), EAT-26 score ≥ 10 (OR: 1.92, 95% CI: 1.26–2.91, *p* = 0.002), BITE score ≥ 10 (OR: 1.45, 95% CI: 1.01–2.08, *p* = 0.045), and BDI-II score ≥ 10 (OR: 3.83, 95% CI: 2.97–4.95, *p* < 0.001). No significant associations were found for gender or sleep duration. To explore potential gender differences, we tested interaction terms between gender and each predictor variable in the logistic regression model. However, none of the interaction terms were statistically significant. These results are shown in Table 5.

## 4. Discussion

This large cross-sectional study of Japanese university students showed that FD was associated with disordered eating behaviors and depressive symptoms. Differences in coping styles were observed between students with and without FD. In multivariable logistic regression analysis, disordered eating attitudes, bulimic symptoms, depressive symptoms and low BMI were significantly associated with FD. These findings suggest that disordered eating behaviors and depressive symptoms may be associated with FD in university students.

In this study, the prevalence of questionnaire-based FD was 6.1% in males and 7.2% in females, which is higher than that reported in a recent global epidemiological study of Japanese participants (2.44%) [5]. While there are several reports of the prevalence of FD, the prevalence varies widely across studies. FD has been reported to be relatively common among young adults in several studies [1,6]. In this study, there was no statistically significant difference in FD prevalence between males and females. Previous studies have reported various results: some have reported higher FD rates in females, whereas others have reported no sex difference [1,6]. These results may reflect differences in sample size, age distribution, and methodological approaches, as well as other factors. University students may experience relatively high levels of academic and psychosocial stress, which has been suggested to influence gastrointestinal symptoms. In addition, dietary patterns common among students, such as irregular meal timing, may also contribute to dyspeptic symptoms. Although it is difficult to compare our results with those of others because their studies included populations of different ages and countries, FD appears to be relatively common among university students.

Students with FD scored higher on both the EAT-26 and BITE and reported higher rates of disordered eating attitudes and bulimic symptoms (clinical or subthreshold levels) than did students without FD. Previous studies have reported a high FD prevalence in patients with EDs [13,56]. Altered eating behavior is closely connected with impaired gastrointestinal sensitivity and motility [13,17], and gastrointestinal symptoms may progress to the critical condition of EDs [19]. These findings suggest that disordered eating patterns may be behavioral correlates of dyspeptic symptoms. FD is increasingly understood within the framework of brain–gut interactions, in which bidirectional communication between the central nervous system and the enteric nervous system modulates gastrointestinal sensation, motility, and mucosal function [11,19]. It is possible that disordered eating behaviors and FD symptoms are interrelated, which may be associated with the persistence and severity of symptoms. Eating pathologies, including both clinical and subthreshold, can lead to impairment, distress, and an increased risk of future onset of depression, substance abuse, health problems, and mortality [57,58]. Our results suggest that disordered eating behaviors and FD symptoms may be interrelated through factors such as gastrointestinal motility, sensory hypersensitivity, postprandial discomfort, and irregular eating patterns, which may be associated with psychological stress and depressive symptoms among university students. Such relationships may contribute to the persistence of symptoms. University students often report high levels of concern about their body image and EDs have increased among university students. Therefore, these relationships may have particular relevance in university settings.

Lower BMI was also associated with FD, particularly among female students. The relationship between BMI and FD is inconsistent, and its underlying mechanism remains unclear. Several studies have reported differences between subjects with and without FD symptoms [1,59,60], whereas others have reported no difference [5,61,62]. A previous study has reported that FD, but not other FGIDs, is associated with being underweight [63]. Studies of patients with FD have reported that weight loss is associated with visceral hypersensitivity and delayed gastric emptying [64], and delayed gastric emptying has also been observed in participants with low BMI [65]. Gastrointestinal discomfort may be associated with restrictive or irregular eating behaviors, while those behaviors may also be related to FD symptoms through altered gut motility and sensitivity. Some studies have reported that the associations between BMI and FD differ according to gender [61]. Although it is difficult to compare our results with those of others because their studies involved respondents of various ages and FD criteria, our results showed that FD may be associated with lower BMI in females. Further research is needed.

Depressive symptoms, including both clinical and subthreshold levels, were more prevalent among students with FD, which is consistent with the findings of previous reports. Subthreshold depressive symptoms are relatively common among adolescents and young adults and may still be associated with psychological distress and functional impairment [45,66,67]. A previous study reported that the prevalence of depression in patients with FD was 32% [68]. Additionally, depression and the severity of FD symptoms are important predictors of quality of life [69]. These results may be related to the gut–brain interaction whereby psychological stress and mood contribute to altered gastrointestinal function and symptom perception. Mood disturbances may affect appetite and eating behaviors, while altered eating behaviors and gastrointestinal discomfort may also be associated with worsening mood. Additionally, university students often experience considerable academic and social stress, which may further contribute to these associations. In this study, we used a BDI-II cutoff score of ≥10 to identify students with depressive symptoms, which allowed us to capture subthreshold depressive symptoms in addition to clinically significant depression. However, the use of this low cutoff may increase the possibility of misclassification, and the associations observed in this study should be interpreted with caution. These findings suggest the need for integrated care approaches that address both gastrointestinal and psychological symptoms, particularly among university students.

With respect to stress coping, students with FD had significantly higher emotion-oriented coping scores. Stress-coping strategies play an important role in reducing stress and somatic problems [31,70]. Emotion-oriented coping reflects emotional responses to a problem, and it is generally regarded as a maladaptive coping style linked to psychological distress [71]. This strategy involves the individual focusing on themselves and their emotional experiences—mainly anger, guilt, and anxiety [25]—which may perpetuate symptom-related distress. FD symptoms themselves may also contribute to a tendency to use emotion-oriented strategies, suggesting a potential bidirectional relationship. Reducing maladaptive coping behaviors may contribute to decreases in depression, anxiety, and stress [72]. This finding suggests that coping styles may be involved in the association between depressive symptoms and FD. Interventions aimed at enhancing adaptive coping strategies may contribute to preventive approaches for FD among university students; however, longitudinal and intervention studies are necessary to confirm their efficacy. In this study, coping styles were examined in relation to FD, but they were not included in the multivariable model because the primary focus of the analysis was on eating behaviors. Further studies are needed to clarify the role of coping styles in FD. Overall, the results of this study can be understood within the framework of multifactorial models of FD and brain–gut interactions.

This study has several limitations that need to be addressed in future research. First, this study employed a cross-sectional design. Therefore, causal relationships and temporal directions between FD and psychological or behavioral factors cannot be determined. Second, FD was identified based on self-reported gastrointestinal symptoms using the Rome IV criteria, without clinical assessment or endoscopic evaluation to exclude structural gastrointestinal diseases. Therefore, some participants classified as having FD may have had other organic causes of dyspeptic symptoms, such as peptic ulcer disease or gastroesophageal reflux disease. This may have resulted in misclassification bias and could have influenced the estimated prevalence and associations observed in this study. In addition, FD subtypes defined by the Rome IV criteria, such as postprandial distress syndrome and epigastric pain syndrome, were not examined separately in this study. The questionnaire assessed the presence of overall FD symptoms rather than specific symptom patterns, and therefore reliable subtype classification was not possible. Future studies using more detailed symptom assessments may help clarify subtype-specific associations. Third, we focused on eating behaviors, depressive symptoms and stress coping, but did not assess other psychological factors such as anxiety. Anxiety has been reported to be an important psychological factor associated with FD and may be strongly linked to it. Because anxiety and depressive symptoms are often correlated, the association observed between depressive symptoms and FD may partly reflect unmeasured comorbid anxiety rather than depression alone. Therefore, the possibility of confounding cannot be excluded, and the observed association should be interpreted with caution. Future studies, including assessments of anxiety, may provide a more comprehensive understanding of the psychological factors associated with FD. Fourth, the response rate was 41% of the total student population, and selection bias due to non-response cannot be excluded. Students who did not complete the questionnaire may have differed from those included in the analysis, for example, with respect to psychological distress or gastrointestinal symptoms. This may have influenced the prevalence estimates and observed associations. Fifth, stress responses may be influenced by an individual’s background, social support, lifestyle habits and environment, which were not evaluated in this study. Sixth, although no significant difference was observed in sleep duration between students with and without FD, previous studies have shown a correlation between sleep disturbance and FGIDs. Future research will need to examine this relationship in more detail. Finally, this study was conducted at a single university in Japan. The generalizability of the findings to other populations may be limited. Future studies should include other universities.

## 5. Conclusions

In university students, FD was associated with disordered eating behaviors and depressive symptoms. Differences in emotion-oriented coping were observed between students with and without FD. These results suggest that integrating assessments of gastrointestinal symptoms, eating behaviors, and psychosocial factors may inform preventive approaches in university health services.

## Figures and Tables

**Table 1:** Results of the annual checkup.

		Students with FD	Students Without FD	*t*
Male		(*n* = 136)	(*n* = 2096)	
Age, years, mean ± SD		20.2 ± 2.4	20.0 ± 2.8	0.80
BMI, kg/m^2^, mean ± SD		20.8 ± 3.2	21.1 ± 3.0	1.04
Sleep duration,				
≤5 h/day	*n* (%)	7 (5.1)	58 (2.8)	
	asr	1.6	−1.6	
6 h/day	*n* (%)	47 (34.6)	789 (37.6)	
	asr	−0.7	0.7	
7 h/ day	*n* (%)	56 (41.2)	918 (43.8)	
	asr	−0.6	0.6	
8 h/ day	*n* (%)	21 (15.4)	293 (14.0)	
	asr	0.5	−0.5	
≥9 h/day	*n* (%)	5 (3.7)	38 (1.8)	
	asr	1.5	−1.5	
Female		(*n* = 151)	(*n* = 1945)	
Age, years, mean ± SD		20.0 ± 1.7	19.8 ± 1.5	1.61
BMI, kg/m^2^, mean ± SD		19.9 ± 2.6	20.4 ± 2.4	2.35 *
Sleep duration				
≤5 h/day	*n* (%)	13 (8.6)	73 (3.8)	
	asr	2.9	−2.9	
6 h/day	*n* (%)	63 (41.7)	850 (43.7)	
	asr	−0.5	0.5	
7 h/ day	*n* (%)	54 (35.8)	781 (40.2)	
	asr	−1.1	1.1	
8 h/ day	*n* (%)	18 (11.9)	215 (11.1)	
	asr	0.3	−0.3	
≥9 h/day	*n* (%)	3 (2.0)	26 (1.3)	
	asr	0.7	−0.7	

SD—standard deviation; BMI—body mass index; asr—adjusted standardized residual. * *p* < 0.05.

**Table 2:** Results of the questionnaire.

	Students with FD		Students Without FD		*t*	*p*	d
	Mean	SD	Mean	SD			
Male	*n* = 136		*n* = 2096				
EAT-26	3.9	(4.8)	2.1	(3.1)	4.17	<0.001 ***	0.53
BITE	5.3	(4.0)	3.4	(3.3)	5.39	<0.001 ***	0.56
BDI-II	9.2	(7.7)	4.8	(6.1)	6.51	<0.001 ***	0.70
CISS-T	55.8	(10.3)	56.1	(11.3)	0.33	0.73	0.03
CISS-E	42.8	(9.9)	40.0	(11.3)	2.87	0.004 **	0.25
CISS-A	43.2	(10.4)	42.4	(11.3)	0.82	0.41	0.07
Female	*n* = 151		*n* = 1945				
EAT-26	4.9	(7.3)	2.5	(3.8)	3.87	<0.001 ***	0.56
BITE	7.5	(6.3)	4.6	(4.5)	5.49	<0.001 ***	0.62
BDI-II	10.8	(9.7)	4.9	(6.2)	7.32	<0.001 ***	0.90
CISS-T	52.7	(11.3)	54.6	(10.6)	2.18	0.02 *	0.18
CISS-E	44.8	(10.7)	39.3	(10.6)	6.05	<0.001 ***	0.51
CISS-A	43.9	(9.8)	44.4	(10.2)	0.50	0.61	0.04

SD—standard deviation; EAT-26—Eating Attitudes Test-26; BITE—Bulimic Inventory Test, Edinburgh; BDI-II—Beck Depression Inventory-II; CISS—Coping inventory for stressful situations. * *p* < 0.05, ** *p* < 0.01, *** *p* < 0.001. Effect sizes are presented as Cohen’s d.

**Table 3:** Frequencies of disordered eating behaviors.

		Students with FD	Students Without FD
Eating attitudes			
Males			
disordered (10 ≤ EAT-26)	*n* (%)	14 (10.3)	66 (3.1)
	asr	4.3	−4.3
non-disordered (EAT-26 ≤ 9)	*n* (%)	122 (89.7)	2030 (96.9)
	asr	−4.3	4.3
Females			
disordered (10 ≤ EAT-26)	*n* (%)	25 (16.6)	114 (5.9)
	asr	5.1	−5.1
non-disordered (EAT-26 ≤ 9)	*n* (%)	126 (83.4)	1831 (94.1)
	asr	−5.1	5.1
Bulimic symptoms			
Males			
bulimic symptoms (10 ≤ BITE)	*n* (%)	16 (11.8)	117 (5.6)
	asr	3.0	−3.0
non-bulimic symptoms (BITE ≤ 9)	*n* (%)	120 (88.2)	1979 (94.4)
	asr	−3.0	3.0
Females			
bulimic symptoms (10 ≤ BITE)	*n* (%)	43 (28.5)	256 (13.2)
	asr	5.2	−5.2
non-bulimic symptoms (BITE ≤ 9)	*n* (%)	108 (71.5)	1689 (86.8)
	asr	−5.2	5.2

EAT-26—Eating Attitudes Test-26; BITE—Bulimic Inventory Test, Edinburgh; asr—adjusted standardized residual.

**Table 4:** Frequencies of depressive symptoms.

		Students with FD	Students Without FD
Depressive symptom			
Male			
depressed (10 ≤ BDI-II)	*n* (%)	64 (47.1)	344 (16.4)
	asr	9.0	−9.0
non-depressed (BDI-II ≤ 9)	*n* (%)	72 (52.9)	1752 (83.6)
	asr	−9.0	9.0
Female			
depressed (10 ≤ BDI-II)	*n* (%)	69 (45.7)	337 (17.3)
	asr	8.5	−8.5
non-depressed (BDI-II ≤ 9)	*n* (%)	82 (54.3)	1608 (82.7)
	asr	−8.5	8.5

BDI-II—Beck Depression Inventory-II; asr—adjusted standardized residual.

**Table 5:** Results of multivariate logistic regression analysis.

	Students with FD (*n* = 287)	Students Without FD (*n* = 4041)	Multivariate Analysis	
	*n* (%)	*n* (%)	OR (95% CI)	*p*-value
Gender (female)	151 (52.6)	1945 (48.1)	1.07 (0.83–1.38)	0.57
BMI (BMI < 18.5 kg/m^2^)	79 (27.5)	764 (18.9)	1.74 (1.31–2.31)	<0.001
Sleep duration (≤6 h/day)	130 (45.2)	1770 (43.8)	1.03 (0.82–1.33)	0.76
Disordered eating attitudes (10 ≤ EAT-26)	39 (13.6)	180 (4.4)	1.92 (1.26–2.91)	0.002
Bulimic symptoms (10 ≤ BITE)	59 (20.5)	373 (9.2)	1.45 (1.01–2.08)	0.04
Depressive symptoms (10 ≤ BDI-II)	133 (46.3)	681 (16.8)	3.83 (2.97–4.95)	<0.001

BMI—Body mass index; EAT-26—Eating Attitudes Test-26; BITE—Bulimic Inventory Test, Edinburgh; BDI-II—Beck Depression Inventory-II; OR—odds ratio.

## Data Availability

All data generated or analyzed during this study are included in this article. Further enquiries can be directed to the corresponding author.

## Abbreviations

The following abbreviations are used in this manuscript:

FD	Functional dyspepsia
FGID	Functional gastrointestinal disorder
DGBIs	Gut–brain interaction disorders
EDs	Eating disorders
EAT-26	Eating Attitudes Test-26
BITE	Bulimic Inventory Test, Edinburgh
BDI-II	Beck Depression Inventory-II
CISS	Coping Inventory for Stressful Situations
BMI	Body mass index
ASR	Adjusted standardized residual
OR	Odds ratio
